# Establishment of a Colorectal Cancer-Related MicroRNA-mRNA Regulatory Network by Microarray and Bioinformatics

**DOI:** 10.3389/fgene.2020.560186

**Published:** 2020-10-23

**Authors:** Dan Jiang, Xiaoliang Xie, Zhenhui Lu, Liyuan Liu, Yuliang Qu, Shan Wu, Yanning Li, Guangqi Li, Hongxia Wang, Guangxian Xu

**Affiliations:** ^1^School of Clinical Medicine, Ningxia Medical University, Yinchuan, China; ^2^Institute of Clinical Laboratory Medicine, Guangdong Provincial Key Laboratory of Medical Molecular Diagnostics, School of Medical Technology, Guangdong Medical University, Dongguan, China; ^3^Department of Colorectal Surgery, General Hospital of Ningxia Medical University, Yinchuan, China; ^4^Department of Hepatobiliary Surgery, General Hospital of Ningxia Medical University, Yinchuan, China

**Keywords:** colorectal cancer, microRNA, mRNA, microarray, bioinformatic analysis

## Abstract

Colorectal cancer (CRC) is one of the most malignant cancers with high morbidity and mortality. MicroRNAs (miRNAs) are small non-coding RNAs that affect biological processes by binding to mRNAs and regulating their expression, and epigenetic alterations including miRNA dysregulation are significantly involved in CRC development. Determining the effect of the miRNA-mRNA network on CRC could be helpful for developing novel therapeutic targets and prognostic biomarkers, and even improving survival. In this study, microarray assays were used to screen differentially expressed miRNAs (DE miRNAs) and mRNAs (DE mRNAs) in CRC and the adjacent normal tissues. Among the detected genes, 42 miRNAs and 142 mRNAs were significantly upregulated in CRC, while 23 miRNAs and 279 mRNAs were significantly downregulated. Through overlapping of predicted targets of DE miRNAs and anti-expressed DE mRNAs, networks of DE miRNAs and DE mRNAs in CRC were established. Additionally, the formation of a protein-protein interaction network of DE mRNAs possibly targeted by DE miRNAs, functional annotation and pathway analysis, stable subnetwork mining, and determination of hub genes provided the probable mechanism used by DE miRNAs and DE mRNAs to regulate CRC growth. Finally, validation of expression and prognostic potential of hub genes provided further support for the results above and indicated that CCL-28, GPR15, PNOC, NUSAP1, and their interacted miRNAs may be a potential signature for prognosis of CRC patients. In sum, we successfully established miRNA-mRNA regulatory networks based on microarray results targeting CRC, and these findings may elucidate the mechanisms used for CRC growth and identify miRNA-related signatures for prognosis and treatment of CRC.

## Introduction

As the third most diagnosed malignancy and the second leading cause of cancer death worldwide, colorectal cancer (CRC) has become a great burden on the healthcare system as well as on all of society (Bray et al., 2018). The development of CRC normally occurs over decades, although recently, the speed of development has increased so that many patients are at the advanced stage upon diagnosis, and even at the metastasizing or incurable stage (Douaiher et al., 2017). Both environmental and genetic factors are involved in the carcinogenesis of CRC, which consists of a complex accumulation of mutations in key regulatory genes and epigenetic regulation (Raskov et al., 2020). Because of a current lack of understanding of the molecular process that occurs in CRC development, there has not been a significant increase in the overall survival rates of patients who have been treated with surgery or cytotoxic and biological therapies (Douaiher et al., 2017). An increase in knowledge regarding the molecular biology of CRC could enable earlier diagnosis, more efficient treatment, and subsequent better prognoses for CRC patients.

Non-coding RNAs, accounting for approximately 98% of the entire genome, were found to be significant contributors to a series of pathological processes, including cancer (Esteller, 2011). MicroRNAs (miRNAs) are small non-coding RNAs whose length is 19–22 nucleotides, and they play important roles in cell growth, differentiation, and survival via completely or incompletely binding to target mRNAs, which results in mRNA degradation or post-transcriptional inhibition (Di Leva et al., 2014). Dysregulated miRNAs often regulate a series of mRNAs involved in functional biological processes. Like other physiological and pathological processes, the initiation and progression of CRC were found to be regulated by a family of miRNAs and their targeted mRNAs. Importantly, previous studies have demonstrated that epigenetic modifiers and alterations including miRNA dysregulation, histone modification, and DNA methylation are involved in CRC pathophysiology, and they could be potent therapeutic targets for CRC (Jung et al., 2020). Thus, exploring functional miRNAs in CRC may be the most convenient and facile method to discover new diagnostic or prognostic biomarkers and therapeutic targets.

Herein, differentially expressed miRNAs (DE miRNAs) and mRNAs (DE mRNAs) in CRC tissues compared with the paired adjacent normal colorectal tissues were screened by microarray assays. Interacting networks were constructed by predicting targets of miRNAs and subsequently overlapping the targeted mRNAs with consistently anti-expressed DE mRNAs. Then, functional annotation, pathway enrichment analysis, and protein-protein interactions (PPIs) were conducted, and hub genes were determined. Moreover, the Gene Expression Profiling Interactive Analysis (GEPIA) database and PrognoScan database were used to validate the expression and prognostic potential of these hub genes (Mizuno et al., 2009; Tang et al., 2017). Finally, a DE miRNA-DE mRNA network highly related to CRC was established, and potential roles and molecular mechanisms were provided. Overall, these findings may provide novel insight into CRC growth and indicate possible miRNA-mRNA-associated mechanisms used for CRC progression.

## Materials and Methods

### Sample Preparation

#### Tissue Specimens

With the approval of the Ethics Committee, three pairs of CRC tumor and adjacent normal tissue samples were collected from patients who underwent surgical treatment at General Hospital of Ningxia Medical University, Yinchuan, Ningxia, China. The clinical characteristic of the three patients were as following: age (63 ± 3 years old); gender (all were male); tumor differentiation (all were moderate); tumor size (4.5 ± 1cm); lymph node metastasis (all were negative); peripheral nerve invasion (1 presence, 2 absence); venous invasion (1 presence, 2 absence); TNM stage (all were in IIB). After snap-freezing in liquid nitrogen, the resected specimens were then stored at −80°C.

#### RNA Extraction and Assessment

The three pairs of pre-frozen tissues were homogenized with TRIzol reagent (Invitrogen, United States) using the Mini-Bead-Beater-16 (Biospec, United States). Then, total RNA was extracted from the mixture using a RNeasy Mini Kit (Qiagen, Germany) with synchronous digestion using DNase (Baseline-ZERO DNase, Epicentre, United States). Subsequently, RNA quantity and quality were evaluated using a NanoDrop ND-1000 spectrophotometer (Thermo Fisher Scientific, United States), and RNA integrity was assessed by denaturing agarose gel electrophoresis.

### Data Analysis

#### Microarray Analysis of miRNAs and mRNAs

Qualified RNA was used in microarray assays that were performed by Kangchen Biotech (Shanghai, China) and conducted with an Agilent miRNA Microarray and Arraystar mRNA Microarray. Briefly, for the miRNA microarray, sample labeling and array hybridization were performed according to the Agilent miRNA Microarray System with the miRNA Complete Labeling and Hyb Kit protocol (Agilent Technology, United States). Additionally, the hybridized arrays were washed, fixed, and scanned using an Agilent Microarray Scanner (part number G2505C). For the mRNA microarray, after removal of rRNA, mRNA was purified from total RNA (mRNA-ONLY^TM^ Eukaryotic mRNA Isolation Kit, Epicenter, United States). Then, each sample was amplified and transcribed into fluorescent cRNA along the entire length of the transcripts without 3′ bias utilizing a random priming method (Arraystar Flash RNA Labeling Kit, Arraystar, United States). Next, 1 μg of each of the labeled cRNAs that was purified by a RNeasy Mini Kit (Qiagen, Germany) was hybridized to the Arraystar human lncRNA Microarray v5.0 that consisted of approximately 21,174 protein coding transcripts. Finally, the hybridized arrays were washed, fixed, and scanned using the Agilent DNA Microarray Scanner.

#### Acquisition of DE miRNAs and mRNAs

Agilent Feature Extraction software (version 11.0.1.1) was used to analyze the acquired array images. Quantile normalization and subsequent data processing were performed using the GeneSpring GX v12.1 software package (Agilent Technologies). After quantile normalization of the raw data, further data analysis was performed for miRNAs and mRNAs that had flags in Detected (“All Targets Value”) for at least one out of six samples. Differentially expressed miRNAs and mRNAs were mined through the following criteria: | Fold change| ≥ 2 and *P* < 0.05. DE miRNAs and DE mRNAs with statistical significance were identified through volcano plot filtering. DE miRNAs and DE mRNAs with “| Fold change| ≥ 2” filtering were displayed by hierarchical clustering that was performed using R scripts.

#### Gene Ontology (GO) Annotation and Kyoto Encyclopedia of Genes and Genomes (KEGG) Pathway Analysis

The Gene Ontology project provides a controlled vocabulary to describe gene and gene product attributes in any organism. To parse their functions, DE mRNAs were mapped to Gene Ontology vocabulary^1^ and KEGG pathways^2^. The *p*-value defined by Fisher’s exact test denotes the significance of GO term enrichment in the DE genes, and the significance of the pathway correlates to the conditions (*p*-value cut-off is 0.05).

#### Prediction of Targets of miRNAs and Construction of the DE miRNA-DE mRNA Networks

MiRwalk is a comprehensive archive supplying the largest collection of predicted and experimentally verified miRNA-target interactions (Sticht et al., 2018). The targets of DE miRNAs were predicted through the miRwalk version 3, and the detailed targets were used in the subsequent analysis. Predicted targets of miRNAs were overlapped with the DE mRNAs, and then the respective networks of upregulated DE miRNAs-downregulated DE mRNAs and downregulated DE miRNAs-upregulated DE mRNAs were constructed and then visualized via Cytoscape software.

#### Protein-Protein Interaction (PPI) Network and Core Subnetwork Analysis

DE mRNAs in the DE miRNA-DE mRNA networks were analyzed by Search Tool for the Retrieval of Interacting Genes/Proteins (string), a bioinformatic tool for analysis of interaction, structure, and effect of proteins. Finally, the PPI network was formed with an interaction score ≥0.4 and then was visualized (von Mering et al., 2003). The Cytoscape plug-in app MCODE was applied to calculate the k-score and filter out the subnetworks (Bader and Hogue, 2003), and genes in the subnetwork with the top k-score were classified as the hub genes. Afterward, the hub genes were entered into String again, and the GO biological process and Reactome pathway analyses were adopted to explain the detailed functions of these hub genes given by the String database.

#### Validation of Hub Gene Expression and Prognostic Potential

The GEPIA database provides fast and comprehensive annotation of gene expression in various cancers. The expression of the hub genes in TCGA (COAD) was validated by GEPIA 2.0 (Tang et al., 2019). Then, their roles in the prognosis of CRC were detected by PrognoScan, a powerful platform for evaluating the biological relationship between gene expression and prognosis.

#### Validation of miRNA-mRNA Interactions

The University of California Santa Cruz (UCSC) Xena browser^3^ were used to download data including expression of mRNA and miRNA in TCGA colon cancer (Haeussler et al., 2019). Through filtering the invalid data, log2 (RPM + 1) of miRNAs and log2 (FPKM + 1) of mRNAs were used to analyze the correlation between miRNA-mRNA pairs in the study.

#### Statistical Analysis

DE miRNAs and mRNAs between two groups were analyzed through paired *t*-tests, and *P* < 0.05 was regarded as statistically significant. Other statistical analyses were performed within the bioinformatic tools mentioned above, and *P* < 0.05 was considered as statistically significant.

## Results

### Profiles of DE miRNAs and DE mRNAs in CRC

**FIGURE 1:**
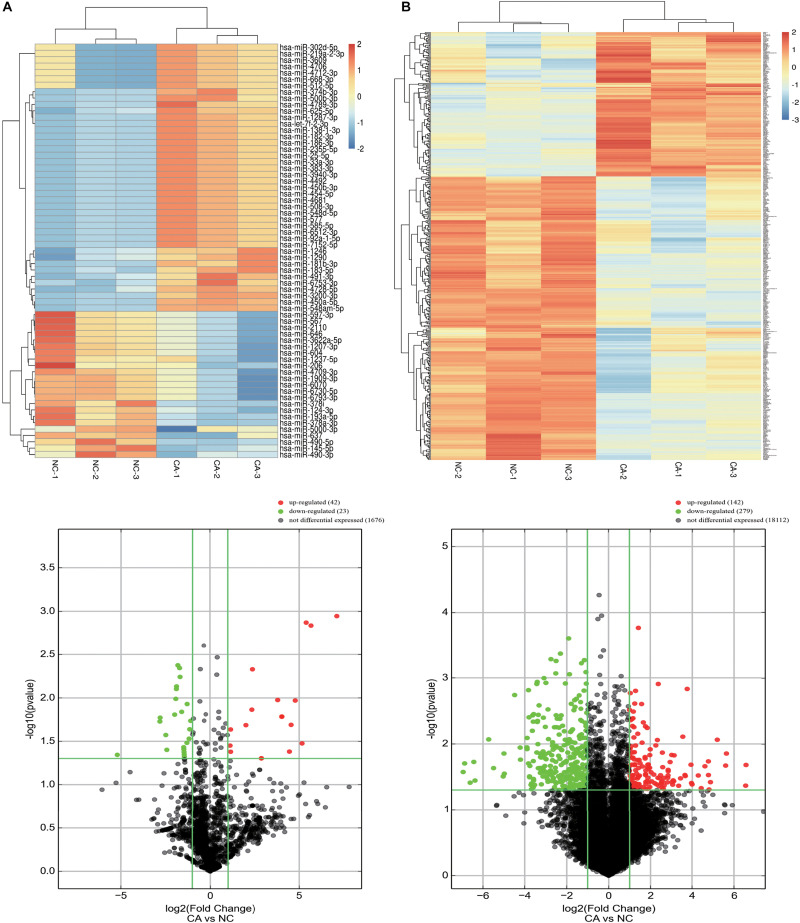
Identification of DE miRNAs and mRNAs in CRC. **(A)** DE miRNAs between CRC cancer and paired adjacent normal tissues; **(B)** DE mRNAs between CRC cancer and paired adjacent normal tissues. Up: heat maps; Down: volcano plots. Differentially expressed miRNAs and mRNAs were mined through the following criteria: | Fold change| ≥ 2 and *P* < 0.05.

The expression of miRNAs and mRNAs between cancer (CA) and normal control (NC) groups was detected by microarray, and variation was assessed using volcano plots and hierarchical clustering analysis. Among the 1,777 miRNAs and 18,533 mRNAs detected, 42 miRNAs and 142 mRNAs were significantly upregulated, and 23 miRNAs and 279 mRNAs were significantly downregulated according to the principle of | fold change| > 2, *P* < 0.05 (Figure 1). All of the expressions of miRNAs and mRNAs detected in groups is shown in Supplementary Table S1.

### Functional Analysis of DE mRNAs in CRC

**FIGURE 2:**
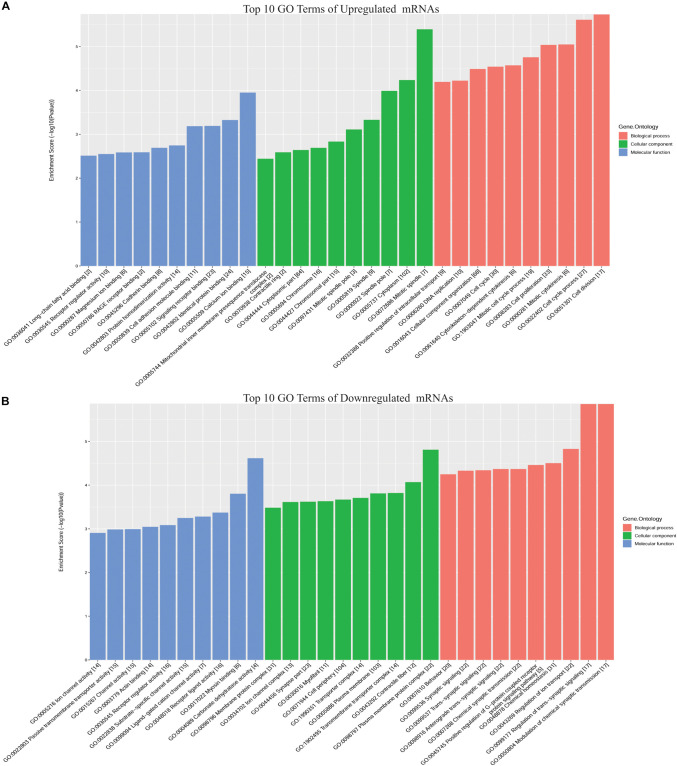
Top 10 GO terms for DE mRNAs in CRC. **(A)** Upregulated DE mRNAs; **(B)** downregulated DE mRNAs. GO annotation includes analyses in biological process (BP), cellular components (CC), and molecular function (MF).

To predict the functions of DE mRNAs in CRC, GO annotation including biological process (BP), cellular component (CC), and molecular function (MF), and KEGG pathway analyses for upregulated and downregulated DE mRNAs were performed (Supplementary Table S2). GO BP analysis revealed that upregulated DE mRNAs were mainly enriched in cell division, the cell cycle process, mitotic cytokinesis, cell proliferation, and the mitotic cell cycle process, while downregulated DE mRNAs were enriched in modulation of chemical synaptic transmission, regulation of trans-synaptic signaling, regulation of ion transport, chemical homeostasis, and positive regulation of G-protein coupled receptor protein signaling pathways. GO CC analysis revealed that upregulated DE mRNAs were enriched in mitotic spindle, cytoplasm, and spindle pole, while downregulated DE mRNAs were enriched in plasma membrane protein complex, contractile fiber, and transmembrane transporter complex. GO MF analysis revealed that upregulated DE mRNAs were enriched in calcium ion binding, identical protein binding, and signaling receptor binding, while downregulated DE mRNAs were enriched in carbonate dehydratase activity, myosin binding, and receptor ligand activity (Figure 2).

**FIGURE 3:**
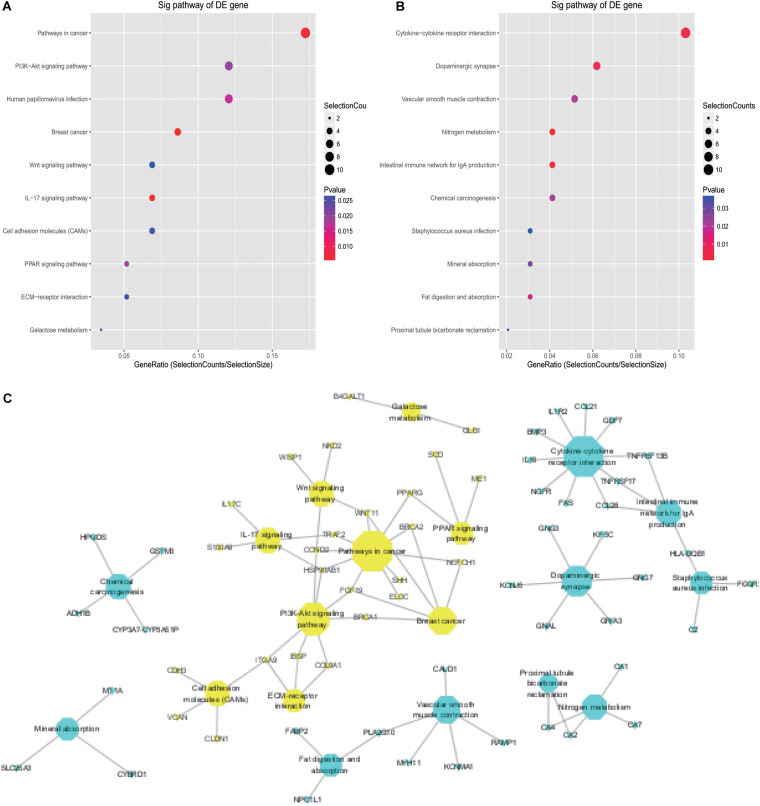
KEGG analysis of DE mRNAs in CRC. **(A)** Top 10 pathways associated with upregulated DE mRNAs; **(B)** top 10 pathways associated with downregulated DE mRNAs; **(C)** detailed information on KEGG pathways associated with DE mRNAs in CRC.

The KEGG pathway analysis revealed that up- and downregulated DE mRNAs were mostly enriched in pathways including pathways in cancer, PI3K-AKT signaling, cytokine-cytokine receptor interaction, and dopaminergic synapse (Figures 3A,B). Detailed information regarding the interactions between the top 20 pathways and associated mRNAs was depicted using Cytoscape, and pathways in cancer and cytokine-cytokine receptor interaction may be the cores of these interactions (Figure 3C).

### miRNA Targets

#### Prediction of Targets of DE miRNAs and DE miRNA-DE mRNA Interaction in CRC

**FIGURE 4:**
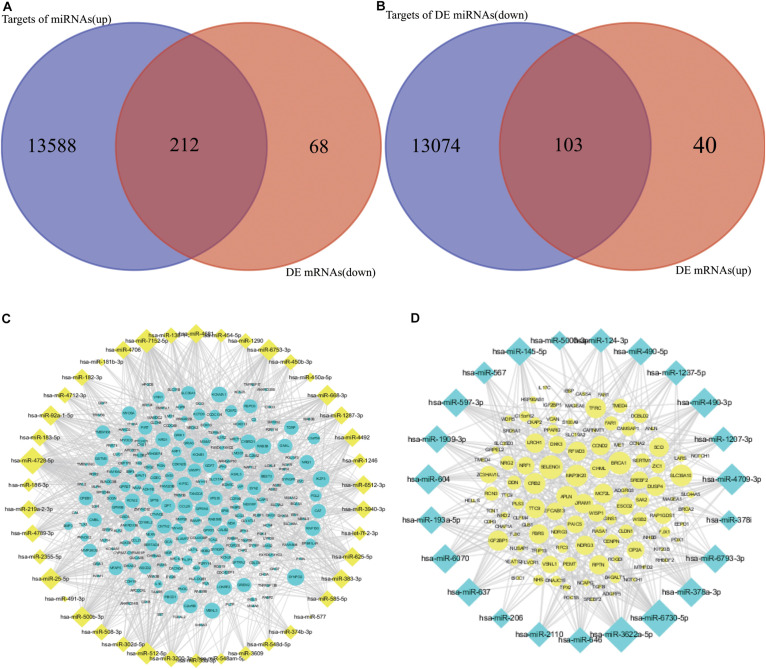
Overlapping of targets of DE miRNAs and anti-expressed DE mRNAs. **(A,C)** Overlapping and network between targets of upregulated DE miRNAs and downregulated DE mRNAs. **(B,D)** Overlapping and network between targets of downregulated DE miRNAs and upregulated DE mRNAs.

MiRNAs usually play roles via binding to the 3′ untranslated region of mRNAs, which results in mRNA degradation or post-transcriptional inhibition. It is viable to illustrate the effect of miRNAs by exploring the reverse relationship between miRNAs and mRNAs. Targets of up- and downregulated DE miRNAs were predicted, and the results indicated that 13,588 and 13,074 mRNAs were potential targets of up- and downregulated miRNAs, respectively (Supplementary Table S3). To construct an effective network between miRNAs and mRNAs in CRC, the target genes and DE mRNAs were overlapped (Figures 4A,B and Supplementary Table S4). Detailed information showing the networks is displayed in Figures 4C,D.

#### PPI Networks of Targeted DE mRNAs of DE miRNAs in CRC

**FIGURE 5:**
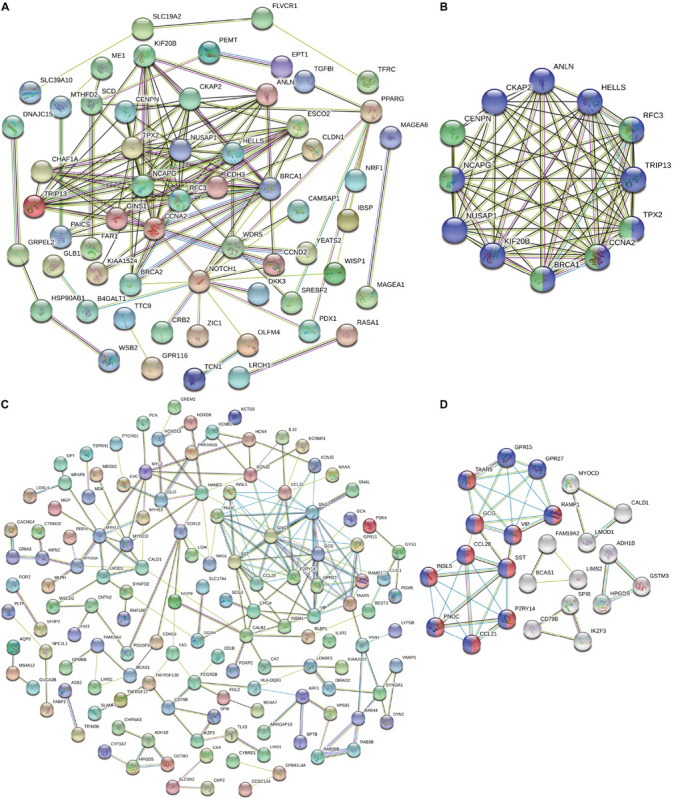
PPI networks consisting of DE networks and stable subnetworks. **(A,B)** PPI and MCODE analysis of upregulated DE mRNAs. **(C,D)** PPI and MCODE analysis of downregulated DE mRNAs. **(A,C)** PPI network analyzed by String; **(B,D)** subnetworks analyzed by MCODE.

To further investigate the functions of genes at the protein level and to reveal the core interactions and mRNAs in the cellular process of CRC, String was used to filter functional genes and to establish a PPI network. Additionally, MCODE in Cytoscape was used to determine critical subnetworks and hub genes. The PPI networks of upregulated DE mRNAs with 59 nodes and 143 edges, and downregulated DE mRNAs with 133 nodes and 215 edges in CRC are shown in Figures 5A,B, respectively. As Figures 5C,D shows, according to a k-score > 2, five critical subnetworks and one critical subnetwork were found in the PPI networks of downregulated and upregulated DE mRNAs in CRC. In accordance with k-score > 5, the respective 12 upregulated and 12 downregulated DE mRNAs were indicated as hub genes with a significant impact on CRC growth (Table 1).

**TABLE 1:** Hub genes in the PPI networks in CRC.

**Group**	**Names of hub genes**
Downregulated candidate genes associated with upregulated miRNAs	P2RY14 SST RAMP1 VIP INSL5 CCL21 PNOC GPR15 GCG GPR27 TAAR5 CCL28
Upregulated candidate genes associated with downregulated miRNAs	CCNA2 NCAPG NUSAP1 HELLS CENPN KIF20B CKAP2 BRCA1 RFC3 ANLN TPX2 TRIP13

### Functional Annotation of Genes in the Important Subnetworks in CRC

**FIGURE 6:**
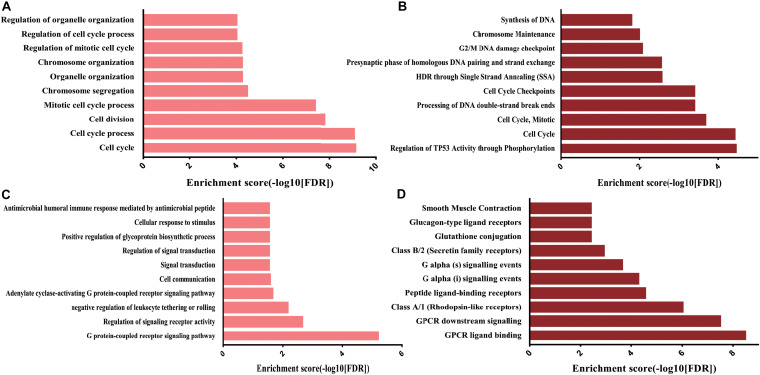
GO BP and Reactome pathway analysis targeting genes of subnetworks. **(A)** Top 10 GO BP of upregulated DE mRNAs; **(B)** top 10 Reactome pathways of upregulated DE mRNAs; **(C)** top 10 GO BP of downregulated DE mRNAs; **(D)** top 10 Reactome pathways of downregulated DE mRNAs.

Subsequently, to clarify the roles of critical subnetworks, the GO biological process and Reactome pathway analyses for proteins in subnetworks were performed via the String database (Supplementary Table S5). The results revealed that for the GO BP analysis, the genes in the subnetwork of upregulated DE mRNAs were significantly enriched in the cell cycle process, while the genes in the subnetwork of downregulated DE mRNAs were significantly enriched in the G protein-coupled receptor signaling pathway and regulation of signaling receptor activity (Figures 6A,C).

For the Reactome pathway analysis, the genes in the subnetwork of upregulated DE mRNAs were significantly enriched in regulation of TP53 activity through phosphorylation and cell cycle regulation, while the genes in the subnetwork of downregulated DE mRNAs were significantly enriched in GPCR ligand binding and GPCR downstream signaling (Figures 6B,D). Interestingly, the most significant genes enriched in the biological processes and pathways (upregulated DE mRNAs: cell cycle; downregulated DE mRNAs: G protein-coupled receptor signaling pathway) were the selected hub genes (Figures 4C,D).

### Identification of Hub Gene Expression and Prognostic Potential in CRC

**FIGURE 7:**
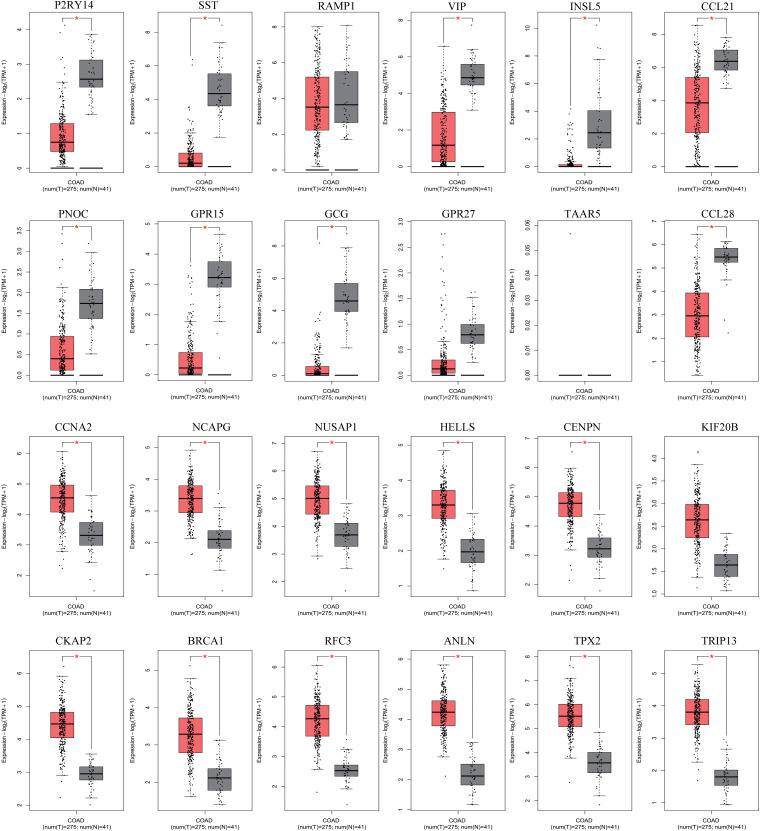
Hub gene expression in the TCGA (COAD) database via GEPIA. *P < 0.05.

**FIGURE 8:**
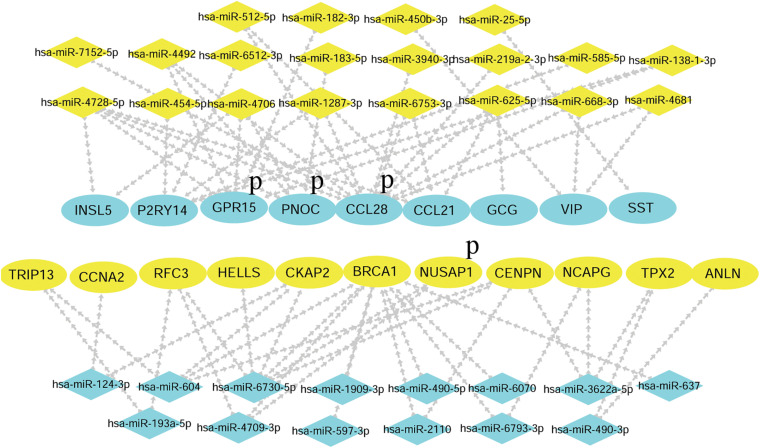
The final networks of candidate miRNAs and hub genes. Blue P indicates that the genes and interacted miRNAs have prognostic potential.

The hub genes selected may affect CRC carcinogenesis from different aspects. To minimize the crisis of the false positives caused by limitation of samples in microarray assays, GEPIA was recruited to identify the expression of hub genes in The Cancer Genome Atlas (TCGA) (COAD) database. As the results showed, among the 12 hub genes in the upregulated miRNA-downregulated mRNAs network, there was lower expression for 9 of 12 genes in the CRC group as compared to the normal tissue group, while there was no significant difference for GPR27, RAMP1, and TAAR5. Among the 12 hub genes in the downregulated miRNA-upregulated mRNA network, 11 of 12 genes were more strongly expressed in the CRC group vs. the Normal group, while there was no significant difference for KIF20B (Figure 7). Thus, the network consisting of 20 upregulated DE miRNAs and 9 downregulated DE mRNAs, and the network consisting of 14 downregulated DE miRNAs with 11 upregulated DE mRNAs was predicted to be the most important miRNA-mRNA interaction in CRC development (Figure 8). The UCSC Xena platform was furtherly used to download data of RNA and miRNA expression in COAD. And through filtering the invalid data and analyzing the correlation between the determined miRNAs and mRNAs, 22 reverse miRNA-mRNA pairs were found according to the criteria of *r* < 0, which was consistent with the results in this study (Table 2). Besides, the prognostic potential of the hub genes was evaluated by the PrognoScan database. A combined analysis of gene expression and the prognostic value of genes revealed higher expression of NUSAP1, which indicated poorer prognosis, while higher expression of CCL28, GPR15, and PNOC resulted in better prognoses in CRC patients (Table 3 and Figure 8). That is, CCL-28, GPR15, PNOC, NUSAP1, and their interacted miRNAs not only affect CRC growth, but also may be a potential signature for prognosis of CRC patients.

**TABLE 2:** The correlation of 22 miRNA-mRNA pairs in COAD.

**miRNA**	**mRNA**	***P*-value**	**Cor value**
hsa-miR-182-3p	GPR15	5.95E−08	–0.25655
hsa-miR-183-5p	PNOC	3.41E−05	–0.1975378
hsa-miR-625-5p	CCL21	0.000122	–0.1834191
hsa-miR-512-5p	CCL21	0.956847	–0.0026049
hsa-miR-4728-5p	CCL21	0.526583	–0.0304769
hsa-miR-450b-3p	VIP	1.61E−06	–0.2279155
hsa-miR-25-5p	SST	0.005465	–0.1331515
hsa-miR-454-5p	P2RY14	2.43E−10	–0.2978361
hsa-miR-490-3p	TPX2	0.003476	–0.1399798
hsa-miR-490-3p	ANLN	0.115051	–0.0757548
hsa-miR-193a-5p	TRIP13	2.21E−08	–0.2645125
hsa-miR-193a-5p	RFC3	0.000578	–0.1645506
hsa-miR-2110	BRCA1	0.280792	–0.0518864
hsa-miR-2110	CENPN	0.059785	–0.0904326
hsa-miR-6730-5p	RFC3	0.06376	–0.0890678
hsa-miR-6730-5p	BRCA1	0.060459	–0.0901959
hsa-miR-6730-5p	CKAP2	0.146241	–0.0698595
hsa-miR-6730-5p	CENPN	0.002615	–0.1441285
hsa-miR-6730-5p	HELLS	0.208851	–0.0604442
hsa-miR-6730-5p	NUSAP1	0.186741	–0.0634947
hsa-miR-4709-3p	RFC3	0.341237	–0.0457923
hsa-miR-6793-3p	BRCA1	0.772704	–0.0139042
hsa-miR-490-5p	BRCA1	0.006446	–0.1305814

**TABLE 3:** Hub genes with prognostic potential and the association between their expression and CRC patient survival.

**Gene name**	**Dataset**	**Endpoint**	**Cohort**	**Probe ID**	**N**	**COX *P*-value**	**HR [95% CI-low CI-upp]**	**Prognosis**
NUSAP1	GSE12945	Overall survival	Berlin	218039_at	62	0.042761	3.84 [1.04–14.12]	Poorer
NUSAP1	GSE12945	Disease-free survival	Berlin	219978_s_at	51	0.01814	34.78 [1.83–660.48]	Poorer
NUSAP1	GSE12945	Overall survival	Berlin	219978_s_at	62	0.000504	34.32 [4.68–251.59]	Poorer
CCL28	GSE17536	Disease-free survival	MCC	224240_s_at	145	0.028541	0.49 [0.25–0.93]	Better
CCL28	GSE14333	Disease-free survival	Melbourne	224027_at	226	0.034875	0.82 [0.68–0.99]	Better
CCL28	GSE14333	Disease-free survival	Melbourne	224240_s_at	226	0.010374	0.66 [0.48–0.91]	Better
GPR15	GSE17537	Disease-free survival	VMC	208524_at	55	0.029864	0.08 [0.01–0.79]	Better
GPR15	GSE17537	Disease-specific survival	VMC	208524_at	49	0.043031	0.08 [0.01–0.92]	Better
PNOC	GSE17536	Disease-specific survival	MCC	205901_at	177	0.02132	0.30 [0.11–0.84]	Better
PNOC	GSE17536	Overall survival	MCC	205901_at	177	0.015574	0.34 [0.14–0.82]	Better

## Discussion

Environmental factors and epigenetic regulation significantly contribute to CRC development (Keum and Giovannucci, 2019). MiRNA dysregulation is a great part of epigenetic regulation, and its expression may change with the alteration of lifestyles, living conditions, or areas that are greatly related to CRC initiation and progression. Analyses that focus on dysregulated miRNAs in CRC patients may provide increased understanding regarding the molecular mechanism of CRC development and more suitable therapeutic views on CRC patient medical intervention (Sapienza and Issa, 2016; Douaiher et al., 2017).

In this study, three pairs of surgical specimens including colorectal cancer and normal para-tumor tissues of CRC patients were collected. Then, differentially expressed miRNAs were detected. According to the principle of | Fold change| ≥ 2 and *P* < 0.05, 42 upregulated miRNAs and 23 downregulated miRNAs were finally determined. Since it is a common practice to control for multiple hypothesis testing, we also tried to adjust the *P*-value for controlling the false discovery rate via Benjamini–Hochberg procedure (Benjamini and Hochberg, 1995). But, the FDR in our study was not chosen to be the criterial because of the poor sensitivity owing to a large FDR, that may be caused by the limitation of the size of the samples (Jung, 2005; Pawitan et al., 2005; Liu and Hwang, 2007). Based on the aim to find some novel miRNA-mRNA interactions that may play roles in CRC development, finally the *P*-value combining with fold change of gene expression was utilized to determine the differentially expressed genes. Furthermore, the differential expression of miRNAs and mRNAs were partly verified in TCGA (COAD) through various methods. It was found that the differential expression of most candidate miRNAs in our results was consistent with the results analyzed by STARBASE in the TCGA (COAD) database (Li et al., 2014). It was verified that a few miRNAs affected CRC growth through different biological processes. For example, miR-625-5p was upregulated and may be an oncogene in CRC (Angius et al., 2019); miR-450b-5p can be induced by oncogenic KRAS and is required for CRC progression (Ye et al., 2016); microRNA-182 can target special AT-rich sequence-binding protein 2 to promote colorectal cancer proliferation and metastasis (Yang et al., 2014); miR-1290 targets INPP48 to contribute to CRC cell proliferation and could be a novel diagnostic biomarker (Ma et al., 2018; Liu et al., 2019b); miR-193a-5p, miR-145-5p, and miR-490-3p restrain cell growth or/and invasion or/and migration via different signaling pathways (Liu et al., 2018; Shirafkan et al., 2018; Niu et al., 2019). Although some explanations have been provided regarding the roles of DE miRNAs in CRC, additional observation and analyses are required for other critical and functional DE miRNAs.

The regulated roles of miRNAs are mainly associated with the posttranscriptional inhibition of targeted coding genes. Studying the functions of their targeted mRNAs and constructing the reversely expressed networks between DE miRNAs and DE mRNAs may provide a preliminary understanding of the mechanism of how DE miRNAs affect CRC. In this study, microarray assays detecting DE mRNAs in the same pairs of tissues finally identified 142 upregulated mRNAs and 279 downregulated mRNAs. GO BP annotation targeting the DE mRNAs indicated upregulated mRNAs were mainly enriched in cell division and cell cycle process while downregulated DE mRNAs were enriched in modulation chemical synaptic transmission and regulation of the G-protein coupled receptor protein signaling pathway.

KEGG pathway analysis demonstrated that DE mRNAs were also enriched in pathways including but not limited to the PI3K-Akt signaling pathway, Wnt signaling pathway, cell adhesion molecules, cytokine-cytokine receptor interaction, nitrogen metabolism, intestinal immune network for IgA production, fat digestion and absorption, and chemical carcinogenesis. Among these pathways, the PI3K-Akt pathway, Wnt signaling pathway, and their crosstalk have been frequently reported to be dysregulated on account of gene mutations or abnormal expression. The subsequently altered functions provide support for our predictions of the functions of DE miRNAs through establishing the DE-miRNA and DE-mRNA networks in CRC (Koveitypour et al., 2019).

There have already been various studies providing ideas about the importance of miRNA-mRNA interacting pairs in CRC from different directions. Zhou et al., obtained 372 pairs of miRNA-mRNA, furtherly analyzed the potential roles in CRC development, and finally determined the significant influence of three miRNAs on the prognosis of CRC patients (Zhou et al., 2015); Mao et al. revealed the post-transcriptional dysregulation of miRNAs in CRC partly through assessing the interactions between miRNAs and alternative polyadenylation (Mao et al., 2020). In this study, overlapping the predicted targets of DE miRNAs with the reversely expressed DE mRNAs, the networks were successfully established of DE miRNAs and DE mRNAs consisting of 42 upregulated DE-miRNAs/212 downregulated DE-mRNAs and 23 downregulated DE-miRNAs/103 upregulated DE mRNAs. STRING and k-score by MCODE in Cytoscape were subsequently used to build a PPI network of DE mRNAs associated with DE miRNAs and locate the central and stable subnetworks. Twelve upregulated and twelve downregulated genes in the critical subnetworks with the highest k-score were finally regarded as the hub genes. Cell cycle process, regulation of TP53 activity through phosphorylation, and the GPCR signaling pathway were mainly involved in the functions of hub genes in CRC. Subsequently, the potential miRNAs and genes were analyzed by comparing with the previous studies. The findings above proved the importance of these genes and also provided valuable clues about the functions of DE miRNAs in CRC.

The GEPIA database also verified the expression of the hub genes in the TCGA (COAD) data. Except for GPR27, RAMP1, TAAR5, and KIF20B, the expression patterns of other hub genes in CRC were consistent between the microarray data and the TCGA (COAD) data in GEPIA. Numerous studies have been performed that support the functions of the candidate hub genes. There was decreased CCL21 expression in CRC and the expression of lymphangiogenic genes, including CCL21, was associated with poor prognosis of both primary and liver metastatic CRC (Mumtaz et al., 2009; Vellinga et al., 2017). RAPTOR facilitated proliferation, migration, and cell cycle progression by promoting the Mtorc1 pathway and transcriptional activation of both URB1 and CCNA2, and CCNA2 can act as a novel biomarker via regulating growth and apoptosis of CRC (Gan et al., 2018; Wang et al., 2020). Moreover, inhibiting the expression of HELLS impaired CRC cell proliferation and induced cell cycle arrest (Liu et al., 2019a). The BRCA1 mutation is not only a cancer susceptibility gene in individuals with CRC or early onset CRC, but it also has great prognostic significance (Garcia et al., 2003; Pearlman et al., 2017; Yurgelun et al., 2017). Overexpression of ANLN was correlated with colorectal cancer progression (Wang et al., 2016). There was higher expression of TRIP13 in CRC, and TRIP13 interacted with YWHAZ, which mediates G2-M transition and epithelial-mesenchymal transition to promote tumor growth (Sheng et al., 2018). Therefore, combining the previous studies and our analysis, the hub genes could be regarded as potential genes affecting CRC development. Based on the preliminary verification of the expression patterns of hub genes in CRC and the predicted DE-miRNA and DE-mRNA networks, potential miRNA-mRNA pairs consisting of 20 upregulated DE miRNAs with 9 downregulated DE mRNAs, 14 downregulated DE miRNAs with 11 upregulated DE mRNAs were constructed. Following that, the correlation between the miRNA-mRNA pairs were examined using the data published previously in TCGA COAD. And hsa-miR-182-3p/GPR15, hsa-miR-183-5p/PNOC, hsa-miR-625-5p/CCL21, hsa-miR-450-3p/VIP, hsa-miR-25-5p/SST, hsa-miR-454-5p/P2RY14, hsa-miR-490-3p/TPX2, hsa-miR-193a-5p/TRIP13, hsa-miR-6730-5p/CENPN, has-miR-490-5p/BRCA1 were verified to be negatively expressed in CRC, partly supporting the effectiveness of the networks. Additionally, experiments in cells and clinical specimens will be focused and performed for further verification.

Evaluating the prognostic significance of these hub genes through PrognoScan may provide additional information regarding the functions of DE-miRNAs and the DE-mRNA network in CRC. Conjoint analysis with different expression and prognostic potential, higher expression of NUSAP1 indicated lower overall survival or disease-free survival rates, while expression of CCL-28, GPR15, and PNOC was positively related to the survival of CRC patients. Thus, the current study constructed the profiles of differentially expressed miRNAs and mRNAs in CRC and the related miRNA-mRNA network. Then, functional annotation discovered the core biological process and pathways, demonstrated the hub genes and effective miRNA-mRNA interaction, and revealed the potential of the hub genes to be prognostic indicators. Finally, the initial mechanism of miRNAs-mRNAs in CRC and their potential as prognostic factors were revealed. It is still necessary to perform further analyses *in silico* as well as additional experiments exploring the targeting functions of the DE miRNAs and mRNA to develop novel mechanisms and therapeutic targets.

## Conclusion

A potential miRNA-mRNA regulatory network that orchestrates the pathogenesis of CRC was constructed, and the functions of the miRNA-mRNA network in CRC were further revealed. These findings indicate the regulatory potential of the miRNA-mRNA network in the development of CRC, and offer possible therapeutic targets and prognostic indicators for CRC.

## Data Availability Statement

The microarray data have been uploaded in NCBI GEO, and the accession numbers are GSE156720 (mRNA microarray data) and GSE156719 (miRNA microarray data).

## Ethics Statement

The studies involving human participants were reviewed and approved by the Ethical Committee of General Hospital of Ningxia Medical University. The patients/participants provided their written informed consent to participate in this study.

## Author Contributions

GX and DJ contributed to the conception and design of the study and developed the methods. DJ, XX, ZL, LL, GL, and SW collected the specimens, extracted RNA, and evaluated the quality of the samples. GX, DJ, and XX discussed the results. DJ, YQ, YL, and HW analyzed the results and wrote the manuscript. All authors contributed to the article and approved the submitted version.

## Conflict of Interest

The authors declare that the research was conducted in the absence of any commercial or financial relationships that could be construed as a potential conflict of interest.

## Footnotes

1 http://www.geneontology.org

2 https://www.genome.jp/kegg/

3 http://xena.ucsc.edu/

## Abbreviations

CRC: colorectal cancer
miRNA: microRNA
DE miRNA: differentially expressed miRNA
DE mRNA: differentially expressed mRNA
PPI: protein-protein interaction
GEPIA: Gene Expression Profiling Interactive Analysis
GO: gene ontology
KEGG: Kyoto Encyclopedia of Genes and Genomes
TCGA: The Cancer Genome Atlas
BP: biological process
CC: cellular component
MF: molecular function.
